# Pregnant women’s perception of midwifery-led continuity care model in Ethiopia: a qualitative study

**DOI:** 10.1186/s12905-023-02456-3

**Published:** 2023-06-08

**Authors:** Ayenew Mose, Yohannes Fikadu, Amare Zewdie, Kassahun Haile, Solomon Shitu, Abebaw Wasie Kasahun, Keyredin Nuriye

**Affiliations:** 1 Department of Midwifery, College of Medicine and Health Science, Wolkite University, Wolkite, Ethiopia; 2 Department of Public Health, College of Medicine and Health Science, Wolkite University, Wolkite, Ethiopia; 3 Department of Medical Laboratory Science, College of Medicine and Health Science, Wolkite University, Wolkite, Ethiopia

**Keywords:** Pregnant women, Perception, Experience, Midwifery-led continuity care, Qualitative study, Ethiopia

## Abstract

**Background:**

A Midwifery-led continuity care (MLCC) model is the provision of care by a known midwife (*caseload model*) or a team of midwives (*team midwifery model*) for women throughout the antenatal, intrapartum, and postnatal period. Evidence shows that a MLCC model becomes the first choice for women and improves maternal and neonatal health outcomes. Despite this, little is known about pregnant women’s perception of the MLCC model in Ethiopia. Therefore, this study aimed to explore pregnant women’s perception and experience of a MLCC model in Ethiopia.

**Methods:**

A qualitative study was conducted in Gurage zone public hospital, Southwest Ethiopia, from May 1^st^ to 15^th^, 2022. Three focused group discussions and eight in-depth interviews were conducted among pregnant women who were selected using a purposive sampling method. Data were first transcribed and then translated from Amharic (local language) to English. Finally, the thematic analysis technique using open code software was used for analysis.

**Results:**

Thematic analysis revealed that women want a continuity of care model. Four themes emerged. Three were specific to women’s improved care. That is, (1) improved continuum of care, (2) improved woman-centred care, and (3) improved satisfaction of care. Theme four (4), barrier to implementation, was concerned with possible barriers to implementation of the model.

**Conclusion:**

The finding of this study shows that pregnant women had positive experiences and showed a willingness to receive midwifery-led continuity care. Woman-centred care, improved satisfaction of care, and continuum of care were identified as the main themes. Therefore, it is reasonable to adopt and implement midwifery-led continuity care for low-risk pregnant women in Ethiopia.

## Background

The World Health Organization (WHO) and the International Confederation of Midwives (ICMs) recommended the midwifery-led continuity care (MLCC) model to scale up quality midwifery care practice [1, 2]. A Midwifery-led continuity care model is the provision of care by a known midwife (*caseload model*) or a team of midwives (*team midwifery model*) for pregnant women throughout the antenatal, intrapartum, and postnatal period [3].

A Midwifery-led continuity care includes continuity of care throughout the childbearing cycle, provision of woman-centred care, and reducing unnecessary medical interventions. A Midwifery-led continuity care model is based on the premise that pregnancy is a normal physiologic process. It is provided in a multidisciplinary network of consultation such as identifying and referring women who require obstetric or other specialist attention [4, 5].

A recent Cochrane review conducted in 2016 showed that the MLCC model reduces the rate of pre-term birth by 24%, neonatal death/ infant death by 19%, regional analgesia by 15%, instrumental birth by 10%, episiotomy by 16%, and increases the rate of spontaneous vaginal birth by 5%. Furthermore, MLCC is cost-efficient, enhances the duration of exclusive breastfeeding, and women’s satisfaction [6, 7]. Therefore, many women value the MLCC model and it becomes their first choice [8]. Despite this, little is known about MLCC in low and middle-income countries including Ethiopia [3, 9].

Globally, 295 000 women died from preventable causes related to pregnancy and childbirth complications in 2017. Of this, 94% occurred in low and middle-income countries [10]. Ethiopia shared the highest burden of maternal and neonatal mortality, with 412 per 100,000 live births and 30 per 1000 live births respectively [11, 12]. A strategy to reduce this burden is largely requested to achieve the 2030 SDGs [13]. In Ethiopia, maternity care is affected by fragmentation in the patient care process, and unnecessary medical intervention. Fragmentation of care results in pregnant women interfacing with many healthcare professionals during their pregnancy, childbirth, and postpartum period [14].

Evidence demonstrates that midwives have shown a willingness to participate in the implementation of the MLCC model in Ethiopia [14, 15]. However, pregnant women’s perception and experience of midwifery-led continuity care is not studied in Ethiopia. Therefore, this study aimed to explore the perception and experience of pregnant women towards midwifery-led continuity care in Ethiopia. The finding of this study is vital for maternal and child health policymakers, hospitals, and stakeholders to design further strategies for upgrading quality midwifery care.

## Method and materials

### Study area and period

This study was conducted in Gurage Zone public hospital. Gurage Zone is located in the southern part of Ethiopia and is found 153 km southwest of Addis Ababa, the capital city of Ethiopia. It has 15 districts and Wolkite town is the administrative centre of the Zone. According to the Central Statistical Agency of Ethiopia in 2007, this zone has a total population of 1 279 646, of whom 622 078 are men and 657 568 are women. The total population of the Gurage zone was projected to be 6.5 million. Gurage zone has a total of 6 hospitals, 72 health centres, and 402 health posts. The study was conducted at Wolkite University Specialized Hospital and Attat hospital which is a service institution of the Catholic Church in Ethiopia. Atat hospital is located about 175 km southwest of the capital, Addis Ababa. The study was conducted from May 1st to 15th, 2022.

### Study approach and selection of study participants

A qualitative study approach was employed to assess the perception and experience of pregnant women regarding midwifery-led continuity care. A purposive sampling method was used to recruit study participants. Eight pregnant women who were present at the hospital during the data collection period were included in in-depth interviews. Twenty-three pregnant women (7, 8, and 8 in each group) were included in the focus group discussion. A midwife who works in the antenatal clinic provided information about the proposed study objective to pregnant women. For FGDs recruitments, we tried to create FGDs with pregnant women who had similar characteristics such as age, gravidity, and educational background. A midwife then invited women to participate in this study. The following questions were used as a guide; (1) what is your experience and perception of maternity care in this hospital? (2) Could you please elaborate particularly related to continuum of care (i.e. antenatal follow-up, childbirth and postnatal care service provision? 3) How do you see receiving healthcare service by one or a team of healthcare professionals particularly (midwives) from pregnancy follow-up up to the postpartum period? 4) Could you tell me more about the advantages and disadvantages of receiving healthcare by one or a team of healthcare professionals? 5) What are the barriers and facilitates of developing and implementing midwifery-led continuity care in this hospital?

Overall, 33 pregnant women were invited to participate in this study. Of this, a total of 31 pregnant women (*n* = 23 FGD and *n* = 8 in-depth interviews) were included with a response rate of 94%. The mean age of study participants was 29.2 (± SD = 4.2) years. Less than half of the participants achieved a secondary level education and half of them were government employees. Regarding gravidity and parity, 4 out of 31 were primigravida and 27 out of 31 were multiparous (Table 1).

**Table 1:** Socio demographic characteristics of study participants in Gurage Zone Hospitals, southwest Ethiopia

Variables	Categories	Frequency	Percentage (%)
Age	20–25	8	25.8
	26–30	19	61.3
	31–40	4	12.9
Educational level	No formal education	4	12.9
	Primary	11	35.5
	Secondary	13	41.9
	College and above	3	9.7
Occupation	Housewife	5	16.2
	Government employee	16	51.6
	Daily labourer	10	32.2
Gravidity	Primigravida	4	12.9
	Multigravida	27	87.1
Parity	Primiparous	4	12.9
	Multiparous	27	87.1

### Data collection procedure

The data were collected through face-to-face in-depth interviews and Focus Group Discussions (FGDs) using a semi-structured guide. The data were recorded using a voice recorder and notes were taken by the investigators. The interviews took from a minimum of 30 min to a maximum of 45 min. The in-depth interview was conducted in a place that was convenient for participants, and where minimum sound disturbance occurred. The interview guide was prepared in English language and translated into Amharic (the local language). To ensure the quality of the data, the guides were reviewed by experts, and a pre-test was done to ensure the flow of the questions and cultural sensitiveness. The principal investigator conducted the in-depth interviews. The data collectors had experience in qualitative research data collection and analysis.

Three FGDs with 7, 8, and 8 pregnant women were conducted to explore the group view as well as their perception and experience of midwifery-led continuity care. The FGDs were conducted in a calm room with minimum sound disturbance and lasted 50 min to one hour. All the FGDs were audiotaped and notes were taken by the authors. The investigators also used simple language and descriptions in conducting the interview.

### Data analysis

Open code version 4.3.1 software of qualitative data analysis for coding and analysis was used. Braun and Clark’s method of thematic analysis was used in the data analysis. Accordingly, we followed six steps of Braun and Clark’s method of thematic analysis. The first step was familiarisation of the data through repeated and active reading of the data set. The next step involves generating initial codes, which helps to organize data at a specific level. The third step is searching for themes through an examination of the coded and collected data extracts. Fourth step includes reviewing of themes to ascertain that each coded data fits properly. The fifth step involves defining and naming each theme and finally, writing up the final analysis and description of findings [16]. The overall process of data analysis used an inductive approach. All audiotaped data from in-depth interviews, FGDs, and field notes were independently transcribed verbatim to Amharic (the local language) after repeatedly listening to the records and then translated into the English language. The translated transcription documents were imported into Open code version 4 3.1 software. After repeatedly reading the transcribed document, the investigators coded and categorized the data into themes and sub-themes. Central themes were constructed based on the natural meaning of the categories. The investigators cross-cheeked the themes, that emerged after analysis, with the respective quotes of the themes. The findings were reported by a detailed description and interpretation of the meanings of the themes. Direct quotes from the participants were also included in the write-up of the findings to provide clear images for readers.

## Results

The findings that emerged from the data analysis of the focused group discussions and in-depth interviews were four themes and nine subthemes (Table 2).

**Table 2:** Topics, theme, and subtheme identified in the data analysis

Topic	Theme	Subtheme
Pregnant women experience and perception of midwifery-led continuity care	1. Improved continuum of care	1.1. Improved antenatal care visit
		1.2. Enhance rate of institutional birth and postnatal care follow up
	2. Improved woman centred care	2.1. Improved women’s awareness about changes during pregnancy
		2.2. Improved maternal healthcare seeking behaviour
		2.3. Build trustful woman–midwife relationships
	3. Improved satisfaction of care	3.1. Satisfaction during pregnancy period
Barriers of implementing midwifery-led continuity care model	1. Healthcare workers skills, knowledge, and availability	1.1. Availability of allocated midwives
		1.2. Occurrences of disagreement between midwives and woman
		1.3. Difference in level of healthcare workers knowledge

### Pregnant women experience and perception of MLCC

In this section, we discussed pregnant women’s perceptions and experiences of midwifery-led care. Three themes emerged. These are improved continuum of care, improved women-centred care, and improved satisfaction of care. We described each subtheme in detail.

### Theme 1. Improved continuum of care

#### Subtheme 1.1. Improved antenatal care visits

The midwifery-led continuity of care is care given to pregnant women during the antenatal, intrapartum, and postnatal period by a primary known midwife or a team of midwives [17]. In this study, the majority of pregnant women stated that receiving all antenatal care visits, delivery, and postnatal service from the same caregiver (midwives) would ensure continuity of information and care.

*‘‘I am reached now 9 months. I have received my pregnancy follow-up from the same midwife. She (the midwife) advised me to give birth in a hospital and not to miss my appointment, and she thought me about the merits of giving birth at a health facility and the demerits of giving birth at home. Additionally, she treats me with respect and dignity.’’ (IDI, PW 1)*

Another study participant stated that receiving care from many healthcare workers in one room was stressful and uncomfortable.

*‘‘...For instance, during my antenatal care visit, there were more than six healthcare workers in one room. Someone asked about my history and took my vital sign, other one did an abdominal examination and requested lab investigations. In those movements, I felt anxious, uncomfortable, and stressed.’’ (IDI, PW 5)*

### Subtheme 1.2. Enhance the rate of institutional birth and postnatal care follow up

According to the 2019 Ethiopian Mini Demographic Health Survey, the rate of institutional birth was low (48%) [12]. Therefore, enhancing institutional birth is crucial to reducing home birth-associated maternal and newborn mortality. The finding of this study showed that midwifery-led continuity care might enhance the rate of institutional birth and postnatal care follow-up. Some of the study participants did not have a positive attitude towards healthcare workers, particularly those who work in the labour and delivery unit.

*‘‘I have heard that sometimes the healthcare workers disrespect and abuse during childbirth. And I believe that as long as I am healthy, why do I come to the hospital? However, the health extension workers advised me to start a follow-up. The care I received here is beyond my expectation. They (midwives) received me warmly and provide health education about preparation for childbirth and the possible complications. I have decided to give my birth here. Even I will inform women who had a negative attitude towards midwives.’’ (IDI, PW 3)*

### Theme 2. Improved woman-centred care

Woman-centred care focuses on the women’s unique needs, expectations, and aspirations; recognizes her right to self-determination in terms of choice, control, and continuity of care; and addresses her social, emotional, physical, psychological, spiritual, and cultural needs and expectations [18]. The midwife-woman relationships are central to the provision of woman-centred care. The result of this study showed that the majority of women revealed that receiving care from the same healthcare workers in antenatal care improved women’s awareness, and healthcare-seeking behaviour, and build a friendly midwife-woman relationship.

### Sub theme 2.1. Enhance women’s awareness of changes due to pregnancy

The finding of this study revealed that receiving care from the same healthcare providers encourage women to ask about any changes during pregnancy and further helps them to have more awareness about the changes that occurred during pregnancy.

*‘‘The healthcare workers have sent me to the maternity waiting home. I have spent more than days here; however, no healthcare worker explains the reason to stay here for such a long time. I am depressed here. Even we don’t know who is responsible for us. Receiving care from an individual midwife would be better because we will build a friendly relationship and have the confidence to ask for further information about the reason why we stay here, the changes during pregnancy, the health of my foetus, and the next care I will receive.’’ (FGD, PW)*

### Subtheme 2.2. Improved maternal healthcare seeking behaviour

Maternal and neonatal mortality is unacceptably high in Ethiopia. The three-delays model proposes that maternal mortality is associated with delays in deciding to seek care, reaching the healthcare facility, and receiving care [19]. The finding of this study showed that receiving care from a known and trusted midwife would improve women’s healthcare-seeking behaviour and at least reduce delays in deciding to seek care.

*‘‘She (midwife) advised me to come to the hospital in the case of rupture of amniotic fluid, decreased foetal movement, and headache. I felt I had developed a headache, and following her counsel I am here for chick up.’’ (FGD, PW)*

### Subtheme 2.3. Build trustful woman–midwife relationships

It is crucial that the relationship between a midwife and a woman is essential for a positive experience for women during the childbirth period. On the contrary, if the relationship is worsened women might experience bad obstetric outcomes. Therefore, the study participants revealed that midwifery-led continuity care could empower women to feel involved in decision-making and exercise choices.

*‘‘…Receiving care from the same health caregiver is comfortable. The same healthcare worker (midwife) can easily understand, and we will build trust and friendship. That in turn, helps us to avoid the fear of asking further information and enable us to be involved in the decision-making process.’’ (FGD, PW)*

On the other hand, midwifery-led continuity care builds trust between the midwife and the pregnant woman, increasing the woman’s confidence in her ability to birth and reducing her fear. The experience of pregnancy, especially in the early stages, differs for women. The stability of women’s relationships and social environment will influence her experience.

*‘‘During my early antenatal care visit, I have been seen by the same midwife. Thus, we became friendly, and I told her all of my history. Later on, on my forth visit, a midwife I know was not available and I felt uncomfortable. I will be grateful if I received all my maternity care from a known and trusted midwife.’’ (FGD, PW)*

This finding demonstrated that receiving care from the same healthcare worker improves consistency of information, enabling women to have and increased sense of choice about their antenatal care, childbirth, and postnatal care with a focus on enabling them to labour and birth with minimal medical intervention. For instance, one study participant stated that she has a known doctor for their previous birth and she feels empowered and has a good relationship with her doctor.

*‘‘I have given birth 3 times at Attat hospital. I am pregnant now four times. In all my previous obstetric history I have received from the same healthcare workers (doctor). She (the doctor) knows everything about my health. Having known healthcare worker is wonderful in all aspects of pregnancy care.’’ (FGD, PW)*

### Theme 3. Improved satisfaction of care

#### Subtheme 3.1. Satisfaction during the pregnancy period

In this subtheme, study participants highlighted midwife counselling regarding daily intake of iron and folic acid supplementation, avoidance of unsubscribed drug intake during the early phase of pregnancy, and unnecessary medical interventions increased their satisfaction of care provided.

*‘‘The health education I received in antenatal care class empowered me to make decisions during the entire period of my pregnancy. A midwife informs me to avoid unnecessary intakes of drugs, to take iron and folic acid supplementation, including immunization, and to avoid unnecessary medical interventions. She (midwife) also told me all the danger signals that occurred during pregnancy and to prepare myself financially, and to decide where to give birth. I am satisfied with all the care I have received from the healthcare workers.’’ (FGD, PW)*

Other study participants stated that healthcare workers’ counselling helped them to alleviate some minor disorders during their pregnancy.

*‘‘I have experienced nausea and vomiting around 8 months of my pregnancy. He (the midwife) advised me to take hard foods frequently and to use large pillows during the night time. That helped me a lot.’’ (IDI, PW 8)*

### Barriers of implementing midwifery-led continuity care

#### Theme 1. Healthcare workers skills, knowledge, and availability

##### Subtheme 1.1. Unavailability of allocated midwives

Study participants mentioned that there are three major barriers to adopting, developing, and implementing midwifery-led continuity care. The first might be the unavailability of allocated midwives due to different reasons such as holidays and transfers to other institutions.

*‘‘Currently, I am near to giving birth. For my next pregnancy, I will choose a known midwife. However, a midwife who is responsible to provide the entire care for me might be absent from the hospital due to different unforeseen reasons. In that case, I might face challenges.’’ (IDI, PW 6)*

##### Subtheme 1.2. Disagreement between midwives and pregnant women

Some of the study participants mentioned that there are different behaviours and personalities of healthcare workers. Study participants mentioned that they might encounter difficulties in case of disagreement or not having a good relationship between the midwife and pregnant woman.

*‘‘…For instance, previously, I followed my pregnancy in a private clinic. There, I smiled, unfortunately. However, the doctor mistreated me. Immediately, I decided to change my follow-up clinic and come to this hospital. A midwife is welcoming and friendly. She (midwife) is a good listener. Sometimes, it might be difficult to continue the entire journey of pregnancy with the same midwife in the case of disagreement like I encountered with the one who works in a private clinic.’’ (IDI, PW 4)*

##### Subtheme 1.3. Differences in the level of healthcare workers knowledge

A handful of study participants stated that they prefer to receive care from different healthcare providers as there are variations in the level of knowledge and skill between healthcare providers.

*‘‘I prefer to receive care from different healthcare providers (midwife and doctors). Because each healthcare provider has a different level of knowledge, skill, and empathy. For example, some healthcare workers have good knowledge while others might have poor knowledge and character. That might affect me from receiving adequate and quality healthcare service.’’ (FGD, PW)*

## Discussion

The current qualitative study aimed to explore pregnant women’s perceptions and experience of midwifery-led continuity care in Ethiopia. The finding of this study revealed that the majority of women have positive views towards developing, introducing, and implementing midwifery-led continuity care. Maternal and infant mortality and mortality rate were high in Ethiopia. Thus, Ethiopia should adopt and implement MLCC Model to enhance the rate of completion of continuum of care including antenatal care, skilled birth attendant, and postnatal follow-up [19].

This study showed that the majority of pregnant women want to have a model of care that can ensure adequate and individualised health counselling and promotion during their antenatal visits which enhances their awareness regarding changes during pregnancy and reduces their fear of childbirth. The finding is in line with a study conducted in Iran indicated that midwife-led care in antenatal clinics improved women’s self-efficacy, the ability of decision-making process, and coping ability during pregnancy [20]. Similar studies showed that midwifery-led care reduces women’s fear of birth and postpartum depression, and it also promotes exclusive breastfeeding [21, 22]. The possible explanation might be receiving care from a known midwife might enhance women’s confidence and trust in midwives [23].

Our study shows that women who had the experience of receiving care from a known midwife had reduced fear of asking questions and more importantly reduced worries and stress. The finding is in line with two studies conducted in Sweden indicating that a known midwife can make a difference in women’s positive childbirth experience [24, 25]. A possible explanation might be that, a positive woman-midwife relationship positively affects the woman’s childbirth experience.

The finding of this study showed that receiving care from similar healthcare workers improves woman centred care and builds a trusting woman-midwife relationship. Similar findings were reported in Ireland and Scotland which showed that midwifery-led care is seen as one important strategy for enhancing women’s choice, decision-making, and sense of involvement in the care process [26, 27]. The possible justification might be known midwives easily recognized women’s social, emotional, physical, spiritual, and cultural needs. Therefore, midwifery-led care can improve woman-centred care including the needs of the baby, the woman’s family, and the community.

In this study, according to women’s experiences, midwife health service provision in an antenatal class increased women’s satisfaction. The finding is in agreement with studies conducted in the Royal Women’s Hospital, Australia which showed that women who had received midwifery-led care had a higher rate of satisfaction and positive experience with their antenatal care follow-up, intrapartum care, and postpartum care [28, 29]. Similarly, studies conducted in Pakistan and China reported the women were satisfied with midwifery-led care [30, 31]. A possible justification for this finding might be that receiving care from a known midwife can reduce waiting time in the clinic; enhance the emotional support for women, thus increasing satisfaction with care. In addition, midwifery-led care addresses the individual woman’s needs like allowing a championship, giving sufficient time for questions, and perinatal counselling on a wide range of issues including labour pain, help with breastfeeding, and post-labour recovery [4, 17].

This study also identified the possible barriers to adopting and implementing midwifery-led continuity care. The absence of an allocated midwife during childbirth emerged as a barrier to developing, adopting, and implementing midwifery-led continuity care. The finding is in line with a study conducted at Bradford Teaching Hospital, England [32]. The possible explanation might be pregnant women might develop fear, stress, and anxiety during the absences of their known midwife. Nevertheless, the model allows a backup midwife who replaces those midwives who are not available at the workplace due to various reasons [4].

Moreover, the occurrence of differences in opinions and /or preferred care options between the allocated midwife and woman can be a barrier. The absence of adequate healthcare workers (midwives) for pregnant women is also mentioned as a barrier to implementing midwifery-led maternity service. The finding is in agreement with a study conducted in a Swiss university hospital [33]. The possible justification might be due to the shortage of midwives in Ethiopia which might pose a serious obstacle to implementing this midwifery-led continuity care.

## Strength and limitation of the study

To our knowledge, this is the first qualitative study conducted in the Ethiopian context to understand women’s perception and experience towards midwifery-led continuity care throughout the antenatal, childbirth, and postpartum period. We identified positive views of pregnant women on the development of the midwifery-led unit. This study also identified the possible barriers to adopting and implementing midwifery-led units in Ethiopia which give insight to stakeholders and policymakers. However, this study has limitations. This study did not include obstetricians, healthcare workers, and stakeholders’ views on the development of midwifery-led continuity care. In addition, data were translated from Amharic language to English language. While English language experts supported the authors to minimize the bias, some meaning may have been lost in translation. Moreover, our findings cannot be generalized due to the small number of participants.

## Conclusion and recommendation

Evidence demonstrates that midwife-led models of maternity care reduce maternal morbidity and mortality. Research also supports midwife-led continuity care models for low-risk pregnant mothers. While maternal mortality and morbidity rates are of concern in Ethiopia, there are limited midwifery-led models of maternity care. Additionally, there is scarce understanding of women’s perceptions of midwife-led care and or the barriers to implementing such a model. Findings from the current study revealed that the majority of participating pregnant women are willing to receive care from a known midwife or team of midwives. It is reasonable therefore, to develop and implement midwifery-led practice for low-risk pregnant mothers in Ethiopia. The barriers to implementation of such a model should be examined however, to facilitate a change of practice in a safe and sustainable means, and improve maternal health outcomes in Ethiopia. The government should support a larger study to explore the possible barriers to implementing a midwifery-led continuity care model across different settings. Based on the findings from this study, we can conclude that women had positive views on the development of a midwifery-led continuum of care in our seating.

## Data Availability

All data generated or analyzed during this study are included in this published article.

## Abbreviations

ANC: Antenatal Care
WHO: World Health Organization
ICMs: International Confederation of Midwife
MLCC: Midwifery-Led Continuity Care
PW: Pregnant Women
FGD: Focused Group Discussion
IDI: In-depth Interview
